# Clinical course & management of childhood nephrotic syndrome in Germany: a large epidemiological ESPED study

**DOI:** 10.1186/s12882-019-1233-1

**Published:** 2019-02-07

**Authors:** Ingo Franke, Malik Aydin, Lisa Kurylowicz, Corinna Elke Llamas Lopez, Rainer Ganschow, Michael J. Lentze, Mark Born

**Affiliations:** 1 Department of General Pediatrics, Division of Pediatric Nephrology, University Children’s Hospital Bonn, Bonn, Germany; 2 Helios University Medical Center Wuppertal, Children’s Hospital, Center for Clinical and Translational Research (CCTR), Center for Biomedical Education and Research (ZBAF), Witten/Herdecke University, Heusnerstr. 40, 42283 Wuppertal, Germany; 3 Department of Radiology, Pediatric Radiology, University Hospital of Bonn, Bonn, Germany

**Keywords:** Primary nephrotic syndrome, Secondary nephrotic syndrome, Childhood, ESPED, Complication

## Abstract

**Background:**

Nephrotic syndrome (NS) is one of the most frequent occurring chronic kidney diseases in childhood, despite its rarely occurrence in the general population. Detailed information about clinical data of NS (e.g. average length of stay, complications) as well as of secondary nephrotic syndrome (SNS) is not well known.

**Methods:**

A nationwide ESPED follow-up study presenting the clinical course and management of children with NS in Germany.

**Results:**

In course of 2 years, 347 children developed the first onset of NS, hereof 326 patients (93.9%) had a primary NS, and 19 patients had a SNS (missing data in 2 cases), the majority due to a Henoch-Schönlein Purpura. Patients with steroid-resistant NS (SRNS) stayed significantly longer in hospital than children with steroid-sensitive NS (25.2 vs. 13.3 d, *p* <  0.001). Patients with bacterial/viral infections stayed longer in hospital (24.9 d/19.5d) than children without an infection (14.2 d/14.9 d; *p* <  0.001; *p* = 0.016). Additionally, children with urinary tract infections (UTI) (*p* < 0,001), arterial hypertension (AH) (*p* < 0.001) and acute renal failure (ARF) (*p <* 0,001) stayed significantly longer in hospital.

Patients with SRNS had frequent complications (*p* = 0.004), such as bacterial infections (*p* = 0.013), AH (*p* < 0.001), UTI (*p <* 0.001) and ARF (*p* = 0.007). Children with a focal segmental glomerulosclerosis (FSGS) had significantly more complications (*p* = 0.04); specifically bacterial infections (*p* = 0.01), UTI (*p* = 0.003) and AH (*p <* 0,001). Steroid-resistance was more common in patients with UTI (*p <* 0.001) and in patients with ARF (*p* = 0.007). Furthermore, steroid-resistance (*p <* 0.001) and FSGS (*p <* 0.001) were more common in patients with AH.

**Conclusions:**

This nationwide, largest German study presents results on the clinical course of children with NS considering a diverse range of complications that can occur with NS. The establishment of a region-wide and international pediatric NS register would be useful to conduct further diagnostic and therapy studies with the aim to reduce the complication rate and to improve the prognosis of NS, and to compare the data with international cohorts.

**Electronic supplementary material:**

The online version of this article (10.1186/s12882-019-1233-1) contains supplementary material, which is available to authorized users.

## Background

Although nephrotic syndrome (NS) is rarely observed in the general population, it is one of the most frequent chronic kidney diseases in childhood. There have been a number of epidemiological studies focusing on childhood NS and it has been shown that the incidence in childhood varies worldwide. In Germany, the reported incidence of NS is 1.2–1.8 per 100,000 children per year [1] in comparison to 3–3.5 per 100,000 children living in Paris and the surrounding area [2] and to 6.49 per 100,000 children per year in Japan [3].

However, only few studies focus on epidemiological data, i.e. clinics at onset, hospital duration and complications in patients with primary (PNS) and secondary NS (SNS). This epidemiological study continues and examines the clinical course and management of childhood NS in our large ESPED (Erhebungseinheit für Seltene Pädiatrische Erkrankungen in Deutschland/ survey unit for rare pediatric diseases in Germany)-based German cohort.

## Methods

We performed an epidemiological study through an ESPED-based survey from 01.01.2005 until 31.12.2006 including all hospitalized children in the age range of 0 to 18 years in Germany with the first onset of NS (hypalbuminuria < 25 g/l, high proteinuria > 40 mg/m^2^/h, accordingly to 1 g/m^2^/d and edema) registered by ESPED.

ESPED received a notification of all patients with NS from each hospital and returned to the reporting hospital a questionnaire regarding the patient’s data, history and any complications (Additional file 1). The questionnaire was completed and sent then back pseudonymously to ESPED in Düsseldorf/Germany. The questionnaire was designed by the Society of Pediatric Nephrology (Arbeitsgemeinschaft für Pädiatrische Nephrologie, APN) in accordance with the German data protection laws. All analyzed data involving human participants were in accordance with the ethical standards and with the 1964 Helsinki declaration and its later amendments or comparable ethical standards. Ethical approval was obtained by the Faculty of Medicine, Rheinische Friedrich-Wilhelms-University, Bonn/Germany Ethics Committee and the study was assigned the human study registration number 081/05. The data extraction and analyzes were performed pseudonymously. Additionally, a telephone survey with pediatricians and nephrologists in the zip code area beginning with 5 in Germany was conducted to identify any possibly missed notification to ESPED [1]. Caregivers provided written informed consent for the patient’s inclusion in the study. All patient’s families gave their written informed consent to their inclusion in the study.

### Statistics

Statistical analyses were conducted using IBM® SPSS version 18.0 (NY, USA). t-tests were used for two independent samples and a One-way ANOVA for more than 2 independent samples was used. The significance level was set at *p* < 0.05. The department of statistics provided epidemiological data used to calculate the incidence of NS. The reported data was given as mean ± Standard Deviation. Comparisons of categorial variables were performed using the Chi-square and Fisher’s Exact tests. Missing values were excluded from the statistical analysis.

## Results

### Primary nephrotic Syndrome

#### Description of patients

347 children were recruited for the present study in the period of 2 years. 326 patients (93.9%) had a primary (idiopathic) NS. Nineteen patients (5.5%) had SNS (for 2 patients the form of NS was unknown). The average age of the study sample was 5.5 ± 3.7 years (*n* = 346, the age for one patient was unknown). 10.5% of the children were of Turkish origin and represents the largest ethnic minority of the study cohort (*n* = 36). Within the study sample, totally 282 patients (81.3%) had a steroid-sensitive nephrotic syndrome (SSNS) and 51 steroid-resistant nephrotic syndrome (SRNS). Twelve patients did not have steroid therapy, for which the reasons are not known and for 2 patients, it is unknown if they received steroid therapy. The incidence of SSNS was 1 per 100,000 and of SRNS was 0.2 per 100,000 children < 18 years in Germany (Table 1) [1].

**Table 1:** Description of demographic parameters (age, gender and ethnicity) of the study cohort, *n* = 347

Primary nephrotic syndrome (PNS)	326
Secondary nephrotic syndrome (SNS)	19
Missing information	2
Male	222
Female	125
Gender ratio	1.8:1
Mean age	5.5 ± SD 3.7 years
Steroid sensitive nephrotic syndrome	282 (Mean age = 5.5 years)
Steroid resistant nephrotic syndrome	51 (Mean age = 6.1 years)
No steroid therapy	12
Ethnicity	
German (PNS/SNS)	215/14
Non-German	109/4
Missing information	4
Origins	
Turkish	36
Russian	8
Others	69

#### Complications of NS

After the onset of NS, one or more complications arose in 106 patients (30.5%). Twenty-eight patients had acute gastroenteritis, 22 patients had a lower airway tract infection (LRTI), 15 patients suffered from acute renal failure (ARF), 34 from hypothyroidism and 34 patients had arterial hypertension (AH) at onset of NS (Table 2).

**Table 2:** Number of individual complications for patients with NS, *n* = 347

Complication	Yes	No	**∑**
Peritonitis	3 (0.9%)	344 (99.1%)	347
Phlegmon	2 (0.6%)	345 (99.4%)	347
Sepsis	3 (0.9%)	344 (99.1%)	347
Lower Respiratory Tract Infection	22 (6.3%)	325 (93.7%)	347
Urinary Tract Infection	8 (6.3%)	339 (97.7%)	347
Tonsillitis	6 (1.7%)	341 (98.3%)	347
Upper Respiratory Tract Infection	12 (3.5%)	335 (96.5%)	347
Gastroenteritis	28 (8.1%)	319 (91.9%)	347
Varizella Zoster Virus infection	3 (0.9%)	344 (99.1%)	347
Hepatitis	1 (0.3%)	346 (99.7%)	347
Acute Kidney Failure	15 (4.3%)	332 (95.7%)	347
Arterial hypertension	34 (9.8%)	313 (90.2%)	347
Hypothyroidism	17 (4.9%)	330 (95.1%)	347
Thrombosis	1 (0.3%)	346 (99.7%)	347
Vasculitis	1 (0.3%)	346 (99.7%)	347

**Fig. 1:**
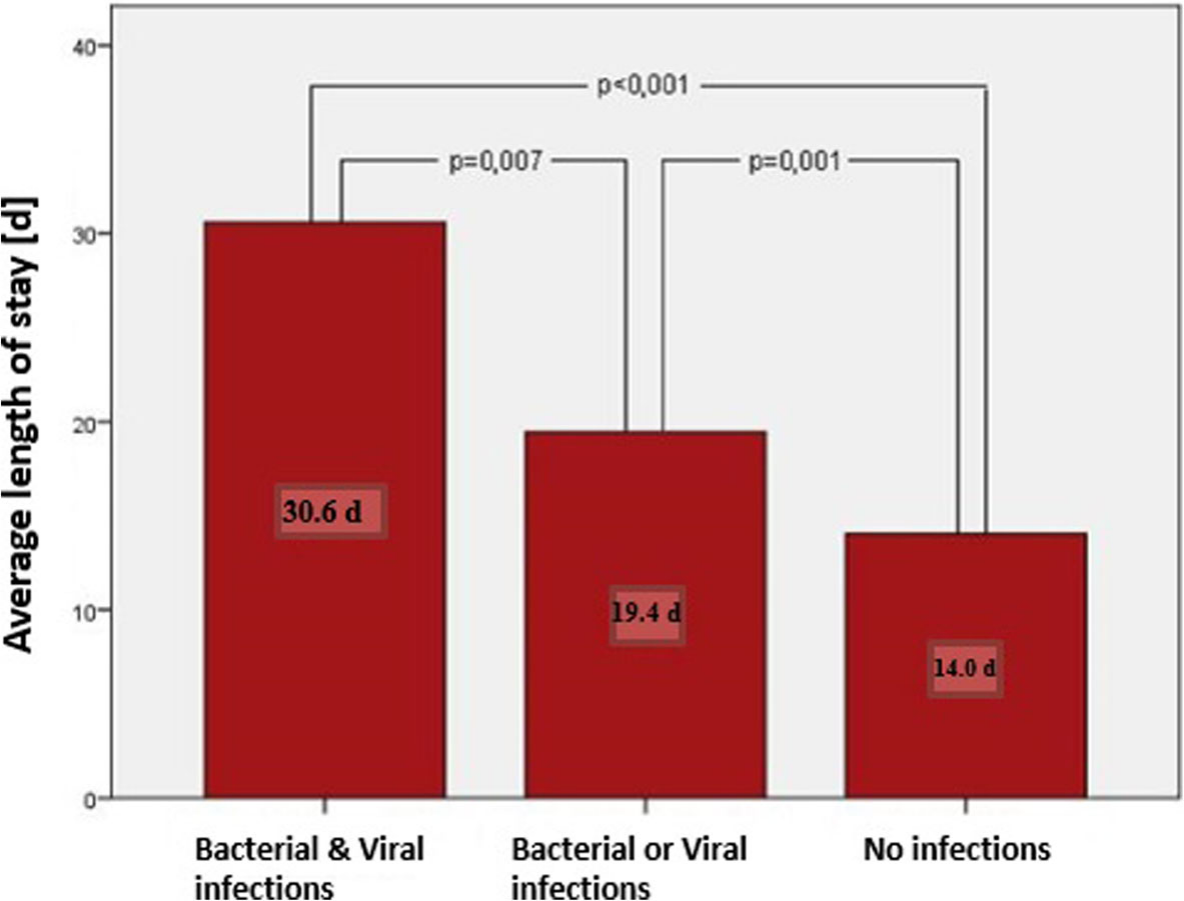
Average length of stay in hospital for children with NS and bacterial and/or viral infections or no infection, *n* = 344

Furthermore, 41 patients had bacterial infections, 40 patients had viral infections and 61 children had viral or bacterial infections. Ten patients had both viral and bacterial infections. The difference in length of stay for patients with viral and bacterial infections, either viral or bacterial versus no viral or bacterial infection was significant (Fig. 1).

The mean age of children with bacterial infections was 4.5 years compared to children with no bacterial infections or no infection at all (5.6 years; *p* = 0.067). The mean age of children with viral infections was 4.8 years compared to children without viral infections (5.6 years).

Patients with a LRTI (average age 4.1 years) were significantly younger than patients without LRTI (5.6 years; *p* = 0.014). LRTIs were less frequent in children of German origin in comparison to children with other ethnic origins (*p* = 0.026). Similarly, children with an urinary tract infection (UTI) were on average 3.3 years old and were younger than children who did not have an UTI (M = 5.6 years; *p* = 0.084). These patients with UTIs had no congenital anomalies of the kidney and urinary tract. Patients with bacterial infections stayed longer in hospital (24.9 d) than children without a bacterial infection (14.2 d; *p* < 0.001). Furthermore, children with viral infections were hospitalized longer (19.5 d) than children without viral infections (14.9 d; *p* = 0.016).

Specifically, children with an UTI (*p <* 0,001), varicella zoster virus (VZV) infection (p = 0,032), ARF (*p <* 0,001) and AH (*p <* 0,001) stayed significantly longer in hospital than children without these complications. Complications occurred more commonly in patients with SRNS than in children with SSNS (*p* = 0.013). In 65% (69 patients, *n* = 106) of cases, children with complications were of German origin, and 33% (35 of 104 patients) of cases were of other ethnic origins and the ethnic origin of 2 (2%) patients was unknown. 67.4% (161 of 239 patients) of German patients had no complications.

Steroid-resistance was more common in patients with UTI (*p* < 0.001) and in patients with ARF (*p* = 0.007). Furthermore, steroid-resistance (*p* < 0.001) and focal segmental glomerulosclerosis (FSGS) (*p <* 0.001) were more common in patients with AH.

Seventy-six (22%, *n* = 343) patients had kidney biopsies, with 31.4% of patients with at least complication and 18.1% of patients without complications having biopsies (*p* = 0.006). Patients with an UTI were more likely to have a histological examination than patients without an UTI (*p* = 0.001). Also, patients with ARF had frequently more kidney biopsies than patients with a normal renal function (*p* = 0.001). Children who were diagnosed with AH at NS onset were also more likely to be biopsied than those patients who had blood pressure values ​​within the normal range (*p* < 0.001). Over half of all patients with AH were biopsied (18/34, 52.9%), whereas 18.8% (58/309) of children without AH were biopsied.

Seven (30%) of the 23 patients with histologically confirmed FSGS had bacterial infections. Ten percent of patients without FSGS had bacterial infections, indicating children with FSGS were more likely to have bacterial infections (*p* = 0.01). More specifically, an increased number of UTIs were diagnosed in patients with FSGS (*p* = 0.008) and, significantly more patients with FSGS had AH (*p <* 0.001) (Table 3).

**Table 3:** Complications arising in children presenting for the first time with Nephrotic syndrome, with and without focal segmental glomerulosclerosis (FSGS), *n* = 342 (missing information for 4 patients)

		FSGS	No FSGS	∑	*p*-value
Peritonitis	No	22 (6.5%)	317 (93.5%)	339	
	Yes	1 (33.3%)	2 (66.7%)	3	0.189
Phlegmon	No	22 (6.5%)	318 (93.5%)	340	
	Yes	1 (50.0%)	1 (50.0%)	2	0.130
Sepsis	No	23 (6.8%)	316 (93.2%)	339	
	Yes		3 (100.0%)	3	1.000
Lower Respiratory Tract Infection	No	20 (6.2%)	300 (93.8%)	320	
	Yes	3 (13.6%)	19 (86.4%)	22	0.176
Urinary Tract Infection	No	20 (6.0%)	315 (94.0%)	335	
	Yes	3 (42.9%)	4 (57.1%)	7	0.008
Upper Respiratory Tract Infection	No	22 (6.7%)	308 (93.3%)	330	
	Yes	1 (8.3%)	11 (91.7%)	12	0.572
Varizella Zoster Virus infection	No	22 (6.5%)	317 (93.5%)	339	
	Yes	1 (33.3%)	2 (66.6%)	3	0.189
Acute Renal Failure	No	22 (6.7%)	305 (93.3%)	327	
	Yes	1 (6.7%)	14 (93.3%)	15	1.000
Arterial hypertension	No	14 (4.5%)	294 (95.5%)	308	
	Yes	9 (26.5%)	25 (73.5%)	34	< 0.001
Hypothyroidism	No	20 (6.2%)	305 (93.8%)	325	
	Yes	3 (17.6%)	14 (82.4%)	17	0.097
Thrombosis	No	23 (6.7%)	318 (93.3%)	341	
	Yes		1 (100%)	1	1.000
Vasculitis	No	23 (6.7%)	318 (93.3%)	341	
	Yes		1	1	1.000

In comparison to patients with minimal-change-glomerulonephritis (MCNS, both biopsy-confirmed or assumed), patients with FSGS had significantly more complications (*p* = 0.04), specifically bacterial infections (*p* = 0.01), UTI (*p* = 0.003) and AH (*p <* 0,001).

Furthermore, ARF was tendentially more frequent in histologically diagnosed MCNS patients than compared to non-biopsied children (*p* = 0.056). Of the 19 patients with SNS, 9 patients had complications, 10 patients had no complications. Of the patients with PNS (*n* = 326), 96 patients had complications and 230 patients had no complications (*p* = 0.099) Three patients with SNS had a tonsillitis, these 3 patients had a bioptically proofed poststreptococcal glomerulonephritis. In total, 6 patients had a tonsillitis. Thus, 50% of the patients with SNS had significantly more tonsillitis than PNS (*p* = 0.003). All other complications, in contrast to patients with PNS, did not occur significantly more or less frequently in patients with SNS (Table 3).

The current guidelines recommend, that children with SSNS should not undergo a biopsy. In our study cohort, a biopsy was conducted in 76 patients (21.9%; *n* = 347). 31 patients had MCNS and 23 patients had FSGS. In addition, 5 patients had diffuse mesangial sclerosis (DMS) and further 5 children had Henoch-Schönlein purpura (HSP). Three patients had histopathologically an IgA-Nephritis; mesangioproliferative GN was diagnosed in 2 patients, and each one patient suffered from an immune complex nephritis, membranoproliferative GN, MGN Type 2 and diffuse proliferative GN. For 3 patients, the histo-pathology is unknown. When patients without biopsies but with SSNS are added, the estimated incidence of MCNS is then 85.5%. Patients who underwent a biopsy were significantly older than patients without the biopsy (6.7 years versus 5.2 years, *p* = 0.011).

The comparison of the average age of patients with MCNS, FSGS, DMS, HSP and IgA nephropathy revealed a significant difference (*p* = 0.002). Children with DMS were averaged 1.3 years younger than those patients with a MCNS (*p* = 0.015), FSGS (*p* = 0.029) and HSP-nephritis (*p* = 0.037). More children beyond the age of 10 years had a histo-pathologically confirmed FSGS compared to children < 10 years of age (p = 0.029). Of the 76 histo-pathologically examined patients, FSGS was commonly more present in girls (*n* = 15) in comparison to boys (*n* = 8; *p* = 0.021). The ALOS was significantly longer for children with FSGS compared to all other patients having NS (27.7 d; *p* = 0.001). Analyzing the data of the biopsied patients, 49 (65.3%) children were steroid-resistant and 22 children were steroid-sensitive (29.3%). 22 patients with FSGS at onset were steroid-resistant (95.7%), only one patient with FSGS (4.3%) initially had SSNS [1].

### Average length of stay

**Fig. 2:**
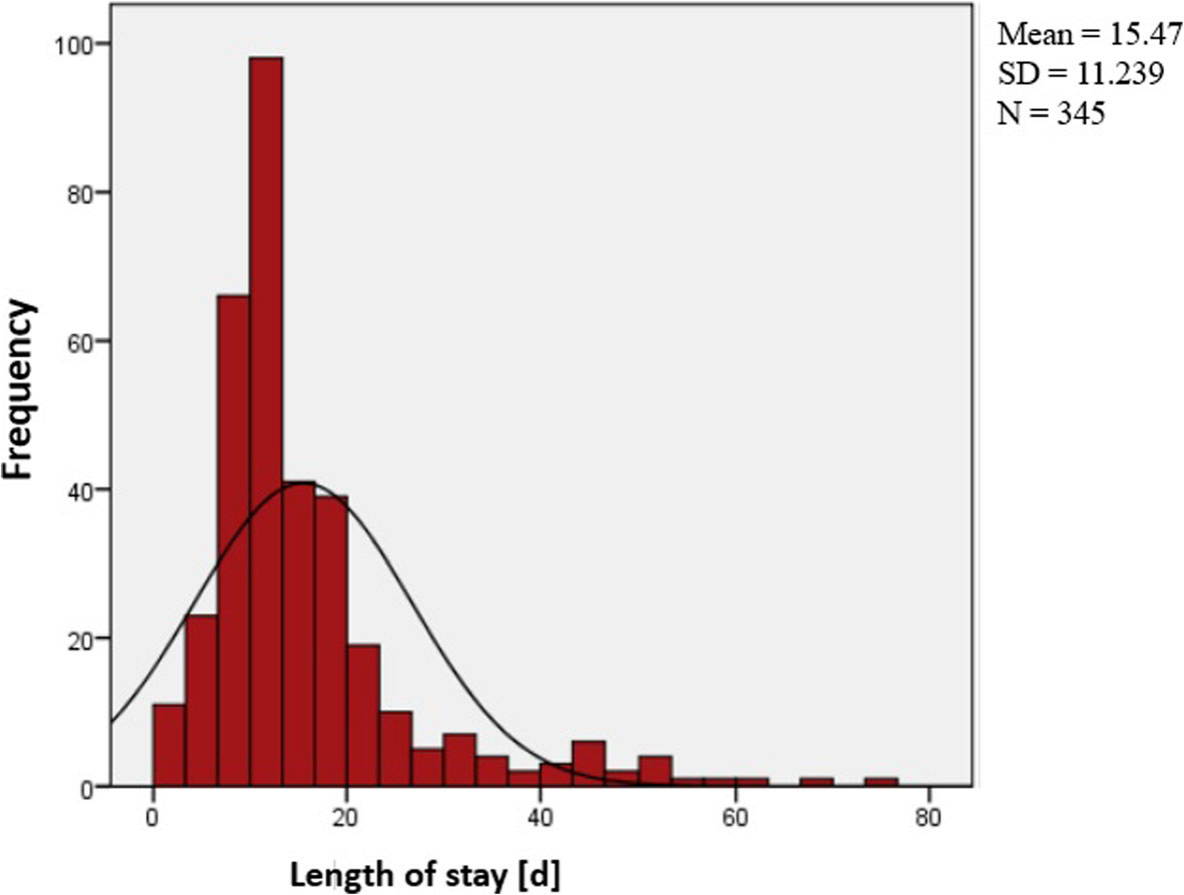
Distribution of length of stay at initial onset of NS, *n* = 345

The Average length of stay (ALOS) was 15.5 ± 11.2 days (Median: 12.0 d) [1]. Twenty-five percent of the children had a length of stay of 9 days and less and 25% were hospitalized for more than 18 days. Seven children were treated as outpatients, or rather daily in-patients (length of stay = 1 day) (Fig. 2). The ALOS of children of 2 years and less was 19.3 d, which is significantly longer than for children aged 3 years and older (14.2 d, *p* = 0.004). Furthermore, the ALOS increased in older children, with children aged 12–14 years staying an average of 16.8 d and children aged 15–18 years staying an average of 20.1 d. A significant difference between the age groups is shown between 0 and 2 years-old and 3–5 years-old (*p* = 0.021), the 0–2-years-old and 6–8-years-old (*p* = 0.003) and the 0–2-years-old and the 9–11-years-old (*p* = 0.022) (Fig. 2). The ALOS in hospital for boys was 14.7 d and 16.8 d for girls (*p* = 0.098). Children with German origins had an ALOS of 16.1 d and children with other ethnic origin was 14.3 d (*p* = 0.135). The ALOS of patients with complications was significantly longer (20.5 days) than for patients without complications (13.2 days; *p* = 0.001). 21.6% of patients with SRNS and 9.5% of patients with SSNS had bacterial infections, with SRNS patients having significantly more bacterial infections than patients with SSNS (*p* = 0.013).

### Secondary nephrotic Syndrome

Nineteen patients (5.5%) had SNS (*n* = 347). Of these, 8 patients developed SNS due to HSP, 5 patients had SNS in the course of an acute streptococcal infection, 3 patients had a glomerulonephritis with a nephrotic component, in 1 patient NS occurred in the realm of Hepatitis C (HCV), in 1 patient due to Epstein-Barr virus, and in 1 patient due to a Parvo-Virus B 19 infection.

The average age of patients with SNS was 6.3 years in comparison to 5.5 years for patients with PNS. Table 4 shows the gender distribution of patients with SNS. 77.8% of patients with SNS were of German origin, 22.2% had other ethnic origins. Comparatively, 66.4% of patients with PNS were of German origin and 33.6% had other ethnic backgrounds.

**Table 4:** Gender distribution of patients with secondary nephrotic syndrome (SNS) & primary nephrotic syndrome (PNS), *n* = 345

	SNS	PNS	∑
Female	4 (21.1%)	121 (37.1%)	125 (36.2%)
Male	15 (77.9%)	205 (62.9%)	220 (63.8%)
**∑**	19 (100.0%)	326 (100.0%)	345 (100.0%)

The ALOS of patients with SNS was 13.8 d, while the children with PNS stayed in hospital an average of 15.6 days. 21.1% of patients with SNS had SRNS, 42.1% had SSNS, 36.8% of patients received no steroids. In comparison, 14.5% of patients with PNS had SRNS, 84.0% had a SSNS, and only 1.5% were not treated with steroids. Significantly more children with PNS were treated with steroids than children with SNS (*p* < 0.001).

Among the patients with PNS, a histological sample was taken from 68 of 323 (21.1%) patients. Five patients with SNS had a histologically confirmed HSP nephritis, 1 patient had an immunocomplex nephritis with Parvo-Virus-B 19 infection, and one patient had a diffuse proliferative glomerulonephritis due to HSP.

## Discussion

Although one of the rarer diseases in children, NS is not unknown among the vast majority of physicians in many countries, including Germany. Literature about the epidemiology and incidence of NS vary, depending on the country and the ethnic origin of the children.

**Fig. 3:**
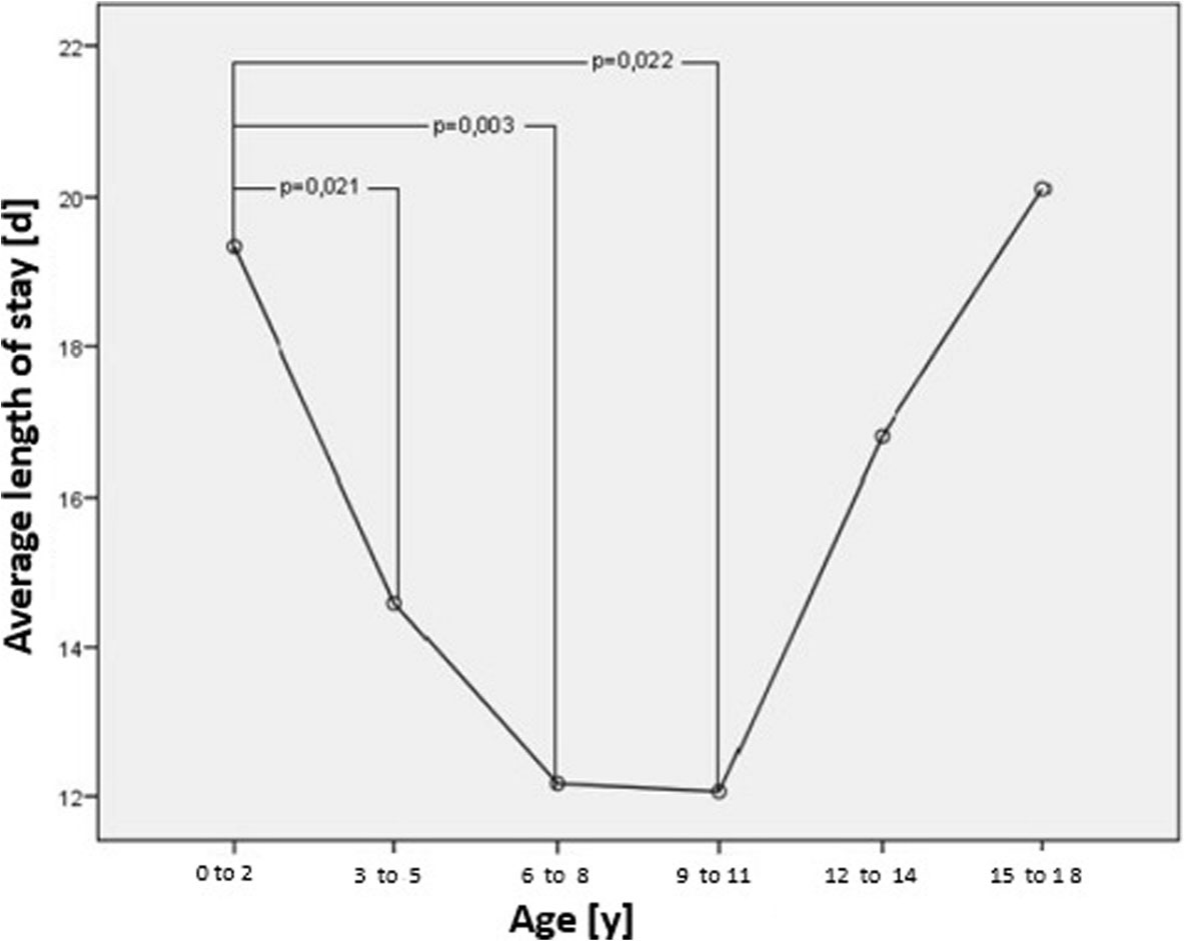
Average length of stay in hospital for patients in different age groups at initial onset of NS, *n* = 344

In the present study, children less than 3 years of age stayed significantly longer in hospital at onset than older children, most likely due to the higher rate of bacterial infections. Interestingly, the ALOS increased for children over 12 years of age (Fig. 3) possibly due to the fact, that the complication rate in this age group is higher compared to the other age groups. Furthermore, the ALOS of girls was 16.0 days slightly compared to boys with 15.0 days, although girls were more likely to have SRNS and FSGS. *Schlesinger* et al. also examined the length of stay in hospital for patients with NS. In comparison, the ALOS was 27.1 days with a median of 19 days. Notably, male patients had an ALOS in hospital twice as long as female patients, and children with dark skin had three times longer hospital stay than children with light skin. Over the past years, there is a trend indicating that the average number of days in hospital decreases [4]. One reason for the increase in the ALOS is that steroid resistance is more present in older aged children. Ethic differences in the ALOS have also been reported [1, 5].

Complications arise for some patients with NS. For 106 patients (30.5%) in the current cohort complications arose, of which 66.3% of the children were of German origins and 33.7% were of other ethnic origins. Ethnic differences in the appearance of onset of SSNS and SRNS or MCNS and FSGS have been found in the literature worldwide, especially between light- and dark-skinned children. Many authors have already shown that African and African-American children were more likely to have FSGS or SRNS than Caucasians [6–9]. Hispanic-American children in New York were more likely to have FSGS than Caucasians and as often as African-American children [7]. Furthermore, in a study by *Bonilla-Felix* et al.*,* Hispanic-American children in Houston had a lower incidence of FSGS than African-American and Caucasian children [6].

The rate of infections also appears to differ across population groups. In a population study from Paris and its surrounding areas, 8% of all patients with NS had bacterial infections that required treatment with intravenous antibiotic therapy. The most frequent infection was peritonitis, followed by pyelonephritis and pulmonary infection [10]. Peritonitis was rarely seen in our study with 0.9%. However, in line with the Paris and surrounding, bacterial infections in patients with SRNS were seen more often than in patients with SSNS. In comparison, in India 39% of patients had infections. The most common infection was upper respiratory tract infection (URTI) (13.7%), followed by lung tuberculosis (10.4%) and peritonitis (9.1%). In a study by *Yap* et al.*,* children with steroid-dependent NS in Singapore were more likely to have an URTI [11] and *Alwadhi* et al. reported that 70.6% of children with NS in India had infections, mostly UTI and empyema [12]. Moreover, *Ibadin* et al. also reported that 44.8% of patients with NS had a UTI [13] however, only 2.3% of our patients in the present study had a UTI. An UTI occurred significantly more frequently in patients with SRNS or FSGS. Children with a LRTI in the present cohort were significantly younger than those without LRTI. The overall trend is that children with bacterial infections are younger. Also, the number of the infections was mostly associated to the number of relapses of NS [14].

Complications in children with intrauterine growth restriction (IUGR) were significantly higher and multiple studies have also reported about the association of SRNS and AH in children with IUGR [15–17]. Children with NS and IUGR were reported to have pneumonia, peritonitis and AH more frequently [15]. Arterial hypertension was observed in 34 of our patients (9.8%), who often had SRNS and FSGS, although no patient in this cohort had IUGR. *Davutoglu* et al. similarly reported that children with AH were frequently more steroid-resistant [14]. Importantly, AH has rarely been observed in children with MCNS [18] but was more common in children with FSGS [19].

Interestingly, hypothyroidism was present in 17 (4.9%) of the cases in the present study. An untreated NS is mostly associated with a hypothyroidism due to the renal loss of TGB, fT3, fT4, T3 and T4 [20]. In total, there is limited literature investigating the relationship between renal disorders and hypothyroidism, with the majority of the literature only describing specific cases. However, in a recent study from India, renal biopsy findings from patients with previously diagnosed hypothyroidism were examined. Of the 16 patients with hypothyroidism, 7 (43.75%) patients had membranous glomerulonephritis (MGN), 4 patients (25%) had both mesangial cell proliferation and MGN. A further 4 cases (25%) had FSGS with chronic interstitial nephritis and only one patient had MCNS. These results also demonstrate hypothyroidism is a common diagnosis presented in patients with NS, in consideration, that 81.25% of patients of the Indian study had NS, and predominantly MGN [13]. It is possible that complicated courses of NS also influence the hormonal status of a patient due to the loss of the thyroid hormones. Further studies are required to establish the relationship between a severe clinical course of NS and hypothyroidism. Thus, physicians should take the thyroid gland into consideration during their diagnostics and therapies of patients with an onset of NS.

In this cohort, ARF was more common in steroid-resistant children than the steroid-sensitive children. At initial onset, 15 patients (4.3%) developed ARF and FSGS did not occur more frequently in these patients. In some studies, this number has been as low in Poland and Nigeria (0.8–1.7%) [21, 22], however in India 17.5% of biopsied patients had ARF at the initial onset of NS [23]. These differences may most likely be explained by the composition of the patient groups.

Furthermore, 22 patients were diagnosed with FSGS and only one patient with FSGS initially had SSNS. It has been previously shown that 75% of steroid-resistant NS patients had FSGS [24, 25]. However, *Nammalwar* et al. reported that 32.9% of all patients with SRNS had FSGS in their study [26].

Naturally, complications can extend the ALOS in hospital. Children with SRNS stayed significantly longer in hospital (25.2 days) than children with SSNS (on average 13.3 days). This may be attributed to the more difficult therapy and the occurring complications observed in patients with SRNS. Except for one child with SRNS (presented with congenital nephrotic syndrome), all steroid-resistant children were biopsied in our study which can also prolong the ALOS.

North American and European literature show that over 90% of children with NS have idiopathic NS [27]. Similar results were found in our study; 93.9% of children had primary NS and 5.5% had SNS, mostly due to HSP. HSP has also been reported to the predominant reason for secondary glomerulonephritis in children in Croatia and Korea [28, 29]]. In most of the cases, HSP nephritis was associated with isolated hematuria, whereas NS was the exception [30, 31]. *Coppo* et al. reported that 35% of children with HSP developed a NS, although only 24.3% adults developed NS. The male to female ratio in children with HSP was 1.44: 1 [32]. *Counahan* et al. reported that a nephritic and nephrotic component seen in HSP were mostly associated with a poorer diagnosis [19]. In the present study, the second most frequent cause of SNS was a streptococcal infection. In the literature, NS is rarely observed in post-streptococcal glomerulonephritis, although proteinuria has frequently been seen [33–35]. A link between the presentation of NS and a poor long-term prognosis has previously been shown [35, 36]. *Al-Rasheed* et al. reported that 60% of patients with bioptically confirmed HSP nephritis had NS, but no patient had a histologically confirmed post-streptococcal GN [35]. *Choi* et al. reported that in Korea that 0.9% of 213 children with NS had a post-streptococcal GN [29] compared to the current data, where approximately 1.8% of patients had poststreptococcal GN.

On the other hand, the pathogenesis of PNS and SNS is very different in Africa. Infectious diseases such as Human Immunodeficiency Virus (HIV), HCV, malaria, syphilis, schistosomiasis and tuberculosis have been reported to be the main causes of NS [37–39]. In South Africa, for example, Hepatitis B virus (HBV) was the most frequent cause of NS [23, 40]. *Buuren* et al. reported that 29 out of 70 children in Namibia with NS were HBV carriers, and 2 patients had syphilis [41]. Interestingly, almost all HBV-positive children showed MGN by histology. In South Africa, the rate of MGN was 20% and mostly associated with HBV; and regional differences reported (e.g. 13.5% in Johannesburg and 40.2% in Durban). FSGS was also common and 40% of FSGS children were previously treated due to a tuberculosis infection and only one patient was HIV positive [42].

In West and East Africa, however, Malaria was commonly associated with NS [43]. Anochie et al. reported that 60% of patients from Nigeria with NS were infected with *Plasmodium falciparum*, two patients had HIV, one had tuberculosis, and one patient had a sickle cell anemia. No patient was HBV positive [44]. In a study by *Eke and colleagues*, 13 of 102 patients with NS had Plasmodium malariae, 9 patients had HBV, and 2 had sickle cell anemia [45]. This number was even up to 38.7% of all children with NS, 13.3% had a sickle cell anemia [46].

Beside the clinical aspects, NS is mostly associated with considerable costs and presents a burden for the health care system. It is particularly characterized by a considerable hospital stay. On average, the costs of one patient with an onset of NS (DRG; ICD-10 for N04.0 – N04.9) are approximately 1200 € (multiplicated with 347 patients = 416,400 €, without complications and longer hospital stay due to other factors in Germany). Thus, depending on the kind of complication, the costs can be significantly higher.

In summary, the present epidemiological study is the largest study of this kind in children with NS with previously performed studies mostly being based on a smaller patient cohort and smaller geographical areas. Briefly, throughout the 2-year duration of the study, data for 347 patients was evaluated. The study provides answers to various epidemiological questions, in particular, regarding SNS, complication rates and the ALOS in relation to patients’ gender, ethnicity and age in Germany. The data is novel in both its quality and quantity. To date, we are not aware of a similar study with a similar cohort (14 million inhabitants < 18 years in Germany). Whilst the study was conducted several years ago, recently published data on a nationwide level and with a large patient cohort regarding NS does not exist, particularly in Germany. Furthermore, this study can also provide a historical insight into the clinical course and management of childhood NS.

## Conclusion

In consideration of the present result, there is a diverse range of complications that can occur with NS. Epidemiological and clinical data of NS in childhood, especially in Germany and in many other countries is limited. Through this large nationwide ESPED-study, novel and interesting data from children with NS in Germany has been gathered and provides a better understanding on NS in childhood and its complications. This study presents a first step for further multicenter studies with extended cohorts to better understand this actually rare but mostly observed nephrological diagnosis in childhood.

## Additional file

Additional file 1: Questionnaire used during the study. (DOCX 16 kb)

## Abbreviations

AH: Arterial hypertension
ALOS: Average length of stay
ARF: Acute renal failure
DGKJ: Deutsche Gesellschaft für Kinder- und Jugendmedizin
ESPED: Erhebungseinheit für Seltene Pädiatrische Erkrankungen in Deutschland
FSGS: Focal segmental glomerulosclerosis
HBV: Hepatitis B virus
HIV: Human Immunodeficiency Virus
HSP: Henoch-Schönlein purpura
LRTI: Lower respiratory tract infection
MCNS: Minimal-change-glomerulonephritis
MGN: Membranous glomerulonephritis
NS: Nephrotic syndrome
PNS: Primary nephrotic syndrome
SNS: Secondary nephrotic syndrome
SRNS: Steroid-resistant nephrotic syndrome
SSNS: Steroid-sensitive nephrotic syndrome
URTI: Upper respiratory tract infection
UTI: Urinary tract infection
