# Supplementary material for: Clinical course & management of childhood nephrotic syndrome in Germany: a large epidemiological ESPED study
Source: BMC Nephrol. 2019 Feb 7;20:45. doi: 10.1186/s12882-019-1233-1 (PMC6367765; doi:10.1186/s12882-019-1233-1)
Supplement: Supplementary file 1 — Questionnaire used during the study. (DOCX 16 kb) [file 12882_2019_1233_MOESM1_ESM.docx]

**Erhebungseinheit für Seltene Pädiatrische Erkrankungen in Deutschland (ESPED)**

**Survey Unit for Rare Pediatric Diseases in Germany**

Research unit of pediatric epidemiology of the German Society of Pediatric and Adolescence Medicine

**First Onset of Nephrotic Syndrome in Childhood**

Dr. I. Franke, MD

Pediatric Nephrology and Metabolic Diseases

University Children’s Hospital

Rheinische Friedrich-Wilhelms-Universität Bonn

53113 Bonn

[ingo.franke@uni-bonn.de](mailto:ingo.franke@uni-bonn.de)

phone: 0228/2873333

fax: 0228/2873444

Reported children’s hospital and contact partner for queries:

Patient’s data

Case n°: Age (at onset): [years], [months]

(given by our study team)

Sex: [ ] male

[ ] female

**Ethnic origin:**

[ ] German [ ] Turkish [ ] Italian [ ] Greek [ ] Polish

[ ] Others [if yes, which country ………]

**Anamnesis:**

Date of admission:

Date of discharge:

**Diagnosis:**

Steroid sensitive nephrotic syndrome [ ] Yes [ ] No

Steroid resistant nephrotic syndrome [ ] Yes [ ] No

(Biopsy) [ ] Yes [ ] No [if yes, which histo-pathology...]

Primary nephrotic syndrome (idiopathic) [ ] Yes [ ] No

Secondary nephrotic syndrome [ ] Yes [ ] No [if yes, which…]

(e.g. SLE, sickle-cell disease, endocarditis etc.)

**Complications:**

Peritonitis [ ] Yes [ ] No

Phlegmon [ ] Yes [ ] No

Other bacterial infections [ ] Yes [ ] No [if yes, which…]

Thrombosis

(venous) [ ] Yes [ ] No [if yes, where…]

(arterial) [ ] Yes [ ] No [if yes, where…]

Other Complications at first onset [ ] Yes [ ] No [if yes, which…]

(e.g. acute renal failure, diarrhea, hypothyroidism etc.)

**We thank you for your cooperation.**
